# Signaling Green: Impact of Green Product Attributes on Consumers Trust and the Mediating Role of Green Marketing

**DOI:** 10.3389/fpsyg.2022.790272

**Published:** 2022-08-02

**Authors:** Kashif Ullah Khan, Fouzia Atlas, Muhammad Zulqarnain Arshad, Sadia Akhtar, Farhan Khan

**Affiliations:** ^1^School of Management Sciences, Ghulam Ishaq Khan Institute of Engineering Sciences and Technology, Swabi, Pakistan; ^2^Department of Management Sciences, Women University Swabi, Swabi, Pakistan; ^3^School of Business Management, College of Business, Universiti Utara Malaysia, Bukit Kayu Hitam, Malaysia; ^4^School of Humanities and Social Science, University of Science and Technology of China, Hefei, China; ^5^Department of Government and Public Policy, National Defense University, Islamabad, Pakistan

**Keywords:** signaling green, green product attributes, consumer trust, green marketing, mediation

## Abstract

The purpose of this research is to highlight the relationship between green product attributes and consumer trust that influence consumers’ decision to purchase green products in the context of Pakistan. This study contributes to determining quantitatively how green product attributes such as physical, perceptual, and reflexive attributes influence consumers’ trust to purchase a green product and investigates the mediating role of green marketing. Data was collected from different industrial sectors through a survey questionnaire. We employed Structural Equation Modeling (SEM) using the SMART-PLS software to check the reliability and validity of the constructs, and to test the hypotheses. This study reveals variations in terms of shaping the sustainable consumers’ buying behavior by modifying product attributes and green marketing strategies that are in congruence with the proposed hypotheses of this study. In the end, the findings and interpretations of the results are given which can guide the managers to develop effective green marketing campaigns in reshaping the purchase intentions of consumers toward their green products.

## Introduction

The novel coronavirus disease (COVID-19) pandemic is deeply affecting humankind around the globe. Its drastic effects are not only limited to public health but has also severely hit national economies and enterprises that are facing a substantial reduction in demand in almost all sectors (Donthu and Gustafsson, 2020; Nicola et al., 2020). Due to the lockdown measures of the government, Pakistani firms are experiencing unprecedented bad impacts on their businesses including, education institutes, small retail markets, transport industry, restaurants, and the hospitality sector (Shafi et al., 2020; Rasheed et al., 2021). The pandemic has also shifted the consumers’ pro-environmental behaviors and attitudes (Peluso et al., 2021). Several researchers asserted the possibility that the pandemic may favor a change in a more healthier direction toward environmentally friendly and sustainable consumption patterns as consumers have become more aware of the humankind’s vulnerability and the relevance of the issues, such as pollution, climate changes, and pandemics, which may have severe effects on both their health and the natural environment (Qi et al., 2020; Mende and Misra, 2021). Customers are becoming increasingly aware of the environmental issues that presently plague the earth’s ecosystems and display a greater propensity toward purchasing from companies whom they perceive as being environmentally conscious (Muduli et al., 2020). Firms showing initiative toward implementing green measures in terms of their present product offerings and manufacturing processes have an advantage in capitalizing on the increased demand for such products (Yadav and Pathak, 2017). When consumers choose to buy products there is a multitude of factors involved in a purchase decision (Zhang et al., 2021). The product is composed of several attributes, and for each product, the attribute is the attached value given by the consumers (Malter et al., 2020).

Burgeoning literature has majorly discussed environmental behavior as an antecedent of intention which is in turn the outcome of environmental attitude and knowledge, yet, far less has been done about the factors that affect the relationship between consumers’ attitude toward going green and green purchase behavior, among the factors that are the attributes of the green product (Cheung and To, 2019). This research study is an endeavor to understand the extent to which the product attributes, physical, perceptual, and reflexive attribute, influence consumers’ trust.

Physical attribute refers to the initial influence of a product on consumers through its appearance, touch, and feel. We all have sensitivities and preferences for particular tastes, designs, colors, facial features, and activities. Perceptual attributes are associated with the functionality of a product, and reflexive attributes are associated with right fullness appraisal and are based on standard-driven emotions such as, how products should be designed and the process of production (Desmet, 2011). Due to rapid industrialization, the growing concerns for the environment have increased for the governments, communities, and consumers (Chiou et al., 2011). Consequent to a paradigm shift in consumers’ intentions and/or preferences to purchase eco-friendly products, the companies’ eco-friendly practices have become the need of the hour (Hosseini and ZiaeeBideh, 2014). The importance of green marketing is an emerging phenomenon as most of the researchers in the field are now focusing on environmentally damaging effects (Dangelico and Vocalelli, 2017). The importance of green marketing is evident from the fact that companies have adopted different environmental approaches that include price, promotion, distribution, and efforts to design products that demonstrate their concern for the environment (Pride and Ferrell, 2008). Due to green marketing, the consumer is becoming more conscious of environmental protection (Groening et al., 2018).

The practical contribution of this study is in the field of sustainability where manufacturing companies pertain to associate with the concept of eco-friendly or corporate social responsibility. This research would enable companies to know what consumers are looking for and how can they win their trust by showing their affiliation/concerns with the environment and the community. No such prior research has tested this model empirically to identify how different important (green) attributes of a product influence green marketing and consumer trust, and the combined effect of green marketing and product attributes on consumer trust. This research, in particular, examines whether or not the green attributes of a product have a significant influence on consumer trust in Pakistan.

This research paper addressed the following research questions:

1. How do green product attributes influence the consumer’s trust to purchase green products?
2. How does green marketing mediate the relationship between green product attributes and consumer trust to purchase green products?

The introduction is followed by a literature review and model with hypothesis development, research methodology, results and analysis, discussion, and implications, and finally, the conclusion is discussed.

## Literature Review and Hypothesis Development

### Physical Factors and Green Marketing

Traditional notions of design engineering have been a multifaceted area of focus for product designers (Shiau et al., 2010), which have required a multitude of theoretical and conceptual models developed to mutually fulfill all performance and aesthetic expectations by prospective buyers. Some of these areas are related to industrial engineering and marketing, which further divide the objectives of each buying stakeholder group (Frischknecht, 2009). For a firm seeking to be environmentally friendly, design considerations must also include material choices that are both affordable and improve the environmental footprint of the product. European businesses based in France and Germany have strived toward the development of “Clean Technologies” which they cite as being enabling factors toward the continuous improvement of green performance in products. Therefore, these physical characteristics may also play a role in governing the marketing campaigns in terms of transferring the cost of environmental protection from the consumer to the industry to facilitate environmental protection within itself (Wen Wan et al., 2017). Chen et al. (2014) have outlined several product design requirements (also known as the 5Es) that they consider as being critical for consideration for consumer requirements and environmental regulations, i.e., the quality of end product, ease of usage, economies of scale and scope, energy-efficiency, and minimal environmental effects.

***H1:** Physical attributes have a positive relationship with green Marketing.*

### Perceptual Factors and Green Marketing

Goldman (1995) propose “Perceptions of products (e.g., that something is beautiful) are what it is noticed from the products.” He suggested eight categories for concepts that describe the perception of products, including, broadly evaluative, formal, evocative, emotional, behavioral, representational, perceptual, and historical. Some of the perceptions in these categories depend upon the experience of the consumer or comparison with other products. Perceptual attributes deal with the phenomenon of people’s judgments about the objective qualities of a product being influenced by design properties (Reid et al., 2010). The process of quantifying the perceptual attributes is difficult, and the only method is “behavioral sciences” that describes how many perceptual attributes are desirable. Existing research highlighted the fact that designers and users perceive perceptual attributes differently (Schnurr et al., 2017). Consumers use subjective attributes for narrowing down their choices when they are presented with many similar alternatives. Product design is an important factor that affects sales, so, designing products that are aligned with perceptual attributes are critical (Landwehr et al., 2011). The ability to integrate green criteria in terms of product design and performance guidelines can be used to enhance the perceptual effects of environmental friendliness on its witnesses and bystanders who see the product in action (Reid et al., 2010).

Past research has supported similar findings of environmentally aware consumers’ willingness to pay more for those products which they deem to be superior in terms of minimizing environmental overheads (Woo and Kim, 2019). There is also a trust-based element in terms of how environmentally aware consumers can be attracted to a particular product concerning its sustainability-oriented attributes, which can be further termed as being that of “Green Trust” (Chen, 2013).

***H2:** Perceptual attributes have a positive relationship with green marketing.*

### Reflexive Attributes and Green Marketing

In reflexive attributes, rationalization and intellectualization of a product occur in the minds of consumers and are subjected to training, education, experiences, and culture of an individual. It is conscious and might be the highest degree of feeling, emotion, and cognition (Norman, 2004). Emotions are functional because they form our position associated with our own environment, attracting us toward certain objects, actions, people, and ideas, and pushing us away from certain others (Frijda, 1986). Satisfying emotions pull us toward the stuff that are (or intended to be) advantageous, whereas negative emotions will drag us away from those that are (or supposed to be) harmful for our welfare (Desmet, 2002).

The first dealing with the product is through the sensory system, which establishes the initial impression or perception about the product. In the next stage, the person appraises the usability of the product, which is dependent on an individual’s culture and experiences. In the last stage, the person reflects on the product and its meaning in relation to him or her, and this is the phase where emotions appear. Emotions at that stage can vary from individual to individual and are dependent on every person’s own situation (Perez Mata et al., 2017).

Organizations communicate their strategies of green marketing with the aim of creating consumers’ attitudes and positive feelings toward the organization (Mercade Mele et al., 2019). Literature has also proven that these attitudes have an influence on customer behavioral outcomes in a diverse manner, such as the intention to purchase or recommend (Pérez and Del Bosque, 2015).

***H3:** Reflexive attributes have a positive relationship with green marketing.*

### Green Product Attributes and Consumer Trust

Green product attributes in recent years have gained increasing attention because of consumers’ eco-friendliness (Sharma and Foropon, 2019). Consumers’ purchase decisions are shifting toward products having green attributes because of their consumption-related environmental problems (Paul et al., 2016). Physical attributes of a product such as size and weight, product quality, design, price, and packaging incline consumer trust in that specific product (Aburumman and Nieto, 2019). Consumers’ trust probably determines their final purchasing decision and this trust is mainly based on the seller’s attitude of not being opportunistic, behaving ethically, and the attributes of the products they sell (Marakanon and Panjakajornsak, 2017). There is a difference between the perception of products perceived by designers and users (Magnier et al., 2019). Consumers when buying a product often make their decision by using subjective reasoning. Consumers also use subjective attributes for narrowing down their choice of the set when they are presented with many similar alternatives, and the same is the case with objective attributes (André et al., 2018). As the design is an important factor that affects sales, designing those products that are aligned with perceptual attributes is most important (Landwehr et al., 2011). The attributes perceived by a customer in a product mainly come from the quality, value, and risk, as concluded in the previous studies (De Medeiros et al., 2016). All these attributes play an important role in the phase of decision-making for that particular product and building long-term purchase trust with it.

The positive image of a product manufacturing firm is sometimes perceived to be a positive attribute to building trust (Nguyen et al., 2019). Attributes perceived by consumers also include country stereotypes and experiences of a product from the country (Perez Mata et al., 2017). The reflexive attribute of an event or product is an appraisal of an event or product as potentially beneficial or harmful for one’s well-being (Scherer, 2001). Appraisal is an assessment of the importance of a stimulus for one’s personal well-being, such as a desire toward purchasing a green product can be appraised as matching with our concern for an environmentally responsible one. However, people belonging to different cultures may appraise the similar (green) product in diverse ways and will experience different emotions regarding its purchase (Desmet et al., 2007). One who is sensitive to environmental issues may respond with the intentions of buying a green product as he/she trusts the green products.

***H4:** Physical attributes have a positive relationship with consumer trust.*
***H5:** Perceptual attributes have a positive relationship with consumer trust.*
***H6:** Reflexive attributes have a positive relationship with consumer trust.*

### Green Marketing and Consumer Trust

Environmental issues have always been the focus of people’s attention, and with the emergence of green consumption, companies are also actively responding to market demands by implementing green marketing (Wang and Kuah, 2018). Companies’ environmental efforts are now being widely regarded as part of their corporate social responsibility, it is all because of the consumers’ awareness regarding environmental responsibility (Nyilasy and Gangadharbatla, 2016).

Jiang and Kim (2015) have proposed that consumers’ understanding of green marketing communications affects their ultimate buying intentions for the business. A strong relationship exists between green marketing and consumer trust because green marketing tools, such as environmental advertisement, eco-labeling, and eco-brand, can earn the consumer’s trust and guide them for purchasing such products (Uddin and Khan, 2018). Therefore, executing a green marketing strategy and sending information about the usage of green products will make consumers feel the advantages of green consumption (for example, the impact on social benefits such as health, the environment, and future generations), which can help consumers to move from intention of purchase to action (Cheung and To, 2019).

***H7:** Green marketing has a positive relationship with consumer trust.*

### Mediating Role of Green Marketing

Today’s consumers are more conscious about the environment than before (Dewald et al., 2014), thus making companies responsible to meet consumers’ demands for eco-friendly (green) products. In the current setting, business companies are using green marketing strategies such as “recyclable and bio gradable” shape to gain a competitive advantage and appeal to environmentally conscious consumers (Szabo and Webster, 2021). Customers not only prefer green products, but they also prefer eco-friendly packaging (Hao et al., 2019). The basic idea behind green marketing is that would-be consumers will see the “greenness” of a good or the service as a gain and shape their buying decisions accordingly. The concept of green marketing strategy has been strengthened with the popularity of terms such as “ozone-friendly” and “recyclability,” which is further extended to include not only the consumer industry but also industrial products and services (Yang and Zhao, 2019).

Thus, to retain loyal customers for long, companies are exploring novel ways to communicate with the customers, such as adopting green marketing. Increased awareness of global warming, climate change, and other environmental issues has prompted eco-friendly manufacturers to produce green products. Consumers respond positively to eco-branded products with environmental features (Chatterjee, 2009). Roberts (1996) investigated the factors that govern environmentally driven consumer consumption patterns and found that consumers’ behaviors are in part due to an internal sense of altruism as well as their willingness to accept responsibility for the ongoing degradation of the environment. The main objective that companies should consider when designing marketing campaigns is the ability to convince consumers that their purchase choices will have an impact on the fate of the ecological health of the planet, thereby empowering them to make environmentally conscious purchase decisions (Schill and Godefroit-Winkel, 2019).

Companies can persuade certain consumers to purchase green products at premium prices that they perceive to be relatively “environmentally friendly” compared to alternative products (Wei et al., 2018). Green marketing campaigns are designed to convince consumers that their purchases play a role in mitigating the ongoing degradation of the environment by way of emphasizing the positive impact they are having in this scenario (Lin and Huang, 2012). Companies are revising their marketing strategies by adopting green marketing standards (including consideration of pricing, promotions, features, and distribution linkages) to meet these needs (Boztepe, 2012).

***H8:** Green marketing mediates the relationship between attributes of a product and consumer trust.*

**FIGURE 1:**
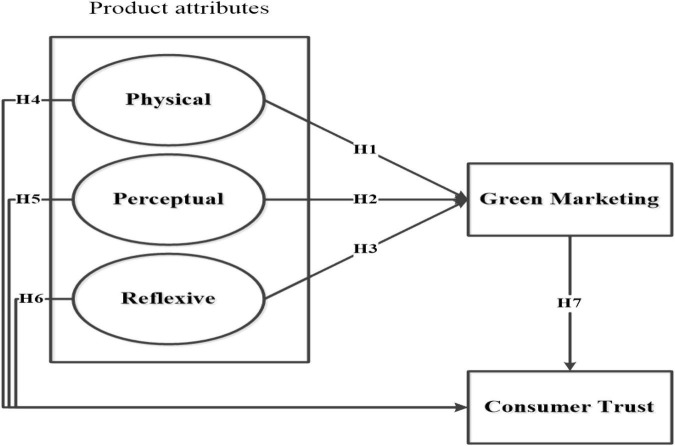
Conceptual framework.

Based on the above hypotheses we propose the conceptual model given in Figure 1.

## Materials and Methods

In this research study, a quantitative approach was employed with the help of a questionnaire due to the reason that data needed to be collected from numerous respondents; therefore, a survey strategy is an efficient way to gather data from a vast population that is perceived to be more reliable. A survey questionnaire was drafted to check the impact of physical, perceptual, and reflexive attributes that respondents associate with to make purchases of a green product, for example a vehicle. The measures were adapted from existing literature and the questions were tailored according to the need of the study in the Pakistani context. The participants (consumers, especially employees in different industrial sectors mentioned in Table 1) were advised to rank items on a five-point Likert scale ranging from (5 = very high to 1 = very low). The data for the survey was collected from different respondents working in different organizations including manufacturing, service, and education all over Pakistan. Questionnaires were distributed to the large population using both offline and online (emails, LinkedIn, WhatsApp, and Facebook) methods. The questionnaire was designed in English and consisted of 24 questions. We received a total of 439 responses out of which 43 questionnaires were discarded because they were not correctly filled. The sample size (*N* = 396) was appropriate to apply the suitable statistical technique to analyze and interpret the collected data. Pilot testing was conducted on the first 75 responses to check the reliability and validity of the data. No changes were required in the questionnaire and the same questionnaire was distributed for further responses. All the respondents were 18 years or older and below the age of 60 years. We choose Structural Equation Modeling (SEM) using SMART-PLS software to check the theory with quantitative methodology.

**TABLE 1:** The frequencies and percentages of personal levels of respondents.

Items	Values	Frequency	Percentage (%)
Gender	Male	249	62.9
	Female	147	37.1
Age	18–25	120	30.2
	26–35	65	33.3
	36–45	132	21.9
	46–55	41	10.4
	Above 55	38	4.2
Monthly income	Under 170$	28	14.5
	171$–278$	14	7.3
	279$–445$	62	31.3
	446$–557$	47	24
	Over 558$	45	22.9
Do you have Children?	Yes	293	74
	No	103	26
Education	Less than or up to High school	82	20.8
	Currently pursuing bachelor’s degree	103	26
	Bachelor’s degree	157	39.6
	Masters and above	54	13.6
Nature of Industry	Manufacturing	132	33.3
	Retail and Service	198	50
	Education	25	6.3
	Other	41	10.4

*Resource: Authors.*

### Data

#### Dependent Variable

Consumer trust is the dependent variable and is measured using two items. These items were adapted and tailored according to the needs of the study from Wang et al. (2015). Since it boosts consumer satisfaction and makes retention easier, consumer trust is a solid predictor of purchase intention (Chen and Barnes, 2007). The Cronbach’s alpha is 0.937 showing excellent construct reliability. The measures are taken from the study of Chen and Chai (2010).

#### Independent Variables

Physical attributes include the size and weight of a product, as well as product quality, design, pricing, and packaging, and all influence customer trust for that specific product (Aburumman and Nieto, 2019). Physical attributes were measured using seven items adapted and modified from Desmet (2010). The measure has been checked for its discriminant validity and reliability, and the Cronbach’s alpha of this scale was 0.890.

Perceptual attributes deal with a phenomenon of people’s judgments of a product’s objective features being impacted by its design properties (Reid et al., 2010). Perceptual attributes were measured using seven items adapted and modified from Desmet (2010). The Cronbach’s alpha for this scale was 0.901. Thus, this scale also achieved reliability and reflected its validity for use.

Reflexive attributes*:* right fullness appraisal is connected with reflexive attributes and is based on standard-driven feelings such as how items should be, their design, and the manufacturing process (Desmet, 2011). Reflexive attributes were measured using five -items adapted and modified from Desmet (2011), and the items were checked for their validity and reliability. The Cronbach’s alpha for this scale was 0.899.

#### Mediating Variable

Green marketing is a comprehensive management strategy that identifies, anticipates, and meets the demands of consumers and society in a profitable and long-term manner (Mercade Mele et al., 2019). Green marketing is the mediating variable and is measured using three items adapted from Mercade Mele et al. (2019). The Cronbach’s alpha is 0.921, which is higher than the cut-off value of 0.7, showing excellent construct reliability.

#### Control Variables

Six items were drafted to collect data about the participants of the study, including gender, age, income, children, education level, and nature of the industry. Descriptive statistics of these variables are shown below in Table 1.

## Results

### Confirmatory Factor Analysis

This analysis refers to an appropriate reliability measurement method for theoretical construct space (Chin and Todd, 1995) by showing the relationship between the observed items and the construct they measure. Table 2 describes the loading of the items used in the survey and shows that all the measures are significant on their path loading, representing acceptable convergent validity.

**TABLE 2:** CFA and t-values.

Items	Measures	Loadings	t-values
Physical Attributes	PAT1	0.758	61.481
	PAT2	0.790	58.045
	PAT3	0.756	58.294
	PAT4	0.761	59.169
	PAT5	0.762	55.603
	PAT6	0.790	56.323
	PAT7	0.758	55.821
Perceptual Attributes	PET1	0.684	44.436
	PET2	0.804	45.291
	PET3	0.864	45.229
	PET4	0.824	46.980
	PET5	0.799	42.647
	PET6	0.810	41.349
	PET7	0.757	40.877
Reflexive Attributes	PER1	0.846	61.469
	PER2	0.860	60.154
	PER3	0.813	60.335
	PER4	0.701	65.909
	PER5	0.668	64.057
Green Marketing	GM1	0.895	58.398
	GM2	0.891	58.737
	GM3	0.842	0.55.159
Consumer Trust	CT1	0.896	54.534
	CT2	0.863	59.934

*Resource: Authors.*

### Data Reliability and Validity

To perform different tests, we first checked the reliability and validity of the data. For this purpose, reliability was checked by Cronbach’s alpha, whereas convergent and discriminant validities were calculated through factor analysis using Smart PLS 3.0, as shown in Table 3. The results show that t Cronbach’s alpha for the constructs ranged from 0.890 to 0.937, showing a high reliability of the model. To measure convergent validity, composite reliability (CR) and mean-variance extracted (AVE) were used, which have a threshold value of 0.7 and 0.5, respectively. Table 3 shows the values of composite reliability (i.e., 0.822–0.864) and AVE (0.515–0.656) greater than the threshold values.

**TABLE 3:** Matrix of construct reliability and validity.

Variables	Cronbach’s alpha (α)	Composite reliability (CR)	Average variance extracted (AVE)
Physical Attributes	0.890	0.864	0.515
Perceptual Attributes	0.901	0.853	0.539
Reflexive Attributes	0.899	0.851	0.656
Green Marketing	0.921	0.830	0.620
Consumer Trust	0.937	0.822	0.614

*Resource: Authors.*

The results confirm that there is no issue of validity i.e., “the degree to which items differentiate between variables” (Thong, 2001, p.152). Therefore, the priority should be a strong correlation within the elements of the same construct. This can be achieved by checking the Fornell–Larcker criterion using correlation analysis and the square root of average variance extracted to inner-construct correlations (Lee et al., 2014). In Table 4, the square root of AVE (bold values in bracket) exceeds the value of the inter-construct. An additional test to validate discriminant validity is determined by the range of values in which the variables lie, that is, they are neither too high (> 0.90) nor too low (< 0.10).

**TABLE 4:** Latent variable correlations and discriminant validity.

	Consumer trust	Green marketing	Reflexive attributes	Perceptual attributes	Physical attributes
Consumer Trust	**(1.000)**				
Green Marketing	0.581	**(0.787)**			
Reflexive Attributes	0.500	0.606	**(0.810)**		
Perceptual Attributes	0.358	0.661	0.734	**(0.743)**	
Physical Attributes	0.443	0.609	0.707	0.718	**(0.791)**

*N = 396, SQRT of AVE is shown in brackets in bold on the major diagonal. Resource: Authors.*

As the reliability and validity of the data are now confirmed, hypothesis testing is done by bootstrapping the results.

### Model Fit Indices

To check a model good fit, the threshold value of chi-square (χ*^2^/df*) should be > 5.

The threshold values for comparative fit index (CFI) and goodness-of-fit index (GFI) should be ≥ 0.9, and normed fit index (NFI) should be ≥ 0.9 (Bentler, 1983, 1988; Browne and Cudeck, 1993). The threshold value for (RMSEA) should be ≤ 0.08 (Jöreskog and Sörbom, 1993; Dudgeon, 2004). The adjusted goodness of fit index (AGFI) should be ≥ 0.8. At last, IFI should be ≥ 0.9 (Bollen, 1989). The structural model of this study shows significant results i.e., χ*^2^*/df is (2), GFI is 0.65, AGFI is 0.89, CFI is 0.97, RMSEA is 0.06, NFI is 0.95, and IFI is 0.97.

### Path Analysis of the Model

To evaluate the structural model, the bootstrapping method was used with 396 samples to find values for T-statistics and to find how significant the variables are. In the path analysis, 4 out of 7 paths were found to be significant whereas the remaining three paths were not significant. Perceptual attributes and reflexive attributes were found to be statistically significant with consumer trust weighting (β = −0.390, *p* ≤ 0.05) and (β = 0.338, *p* ≤ 0.05), respectively. Perceptual attributes were also found to be statistically significant with green marketing (β = 0.375, *p* ≤ 0.05). Green marketing was found to be statistically significant with consumer trust weighting (β = 0.512, *p* ≤ 0.05). This implies that hypotheses H2, H3 H5, and H7 are supported.

Physical attributes are not statistically significant with consumer trust (β = 0.201, *p* = 0.156) and green marketing (β = 0.163, *p* = 0.164). Reflexive attributes were not statistically significant with green marketing weighting (β = 0.212, *p* = 0.075). Thus, this proves that hypotheses H1, H4, and H6 are not supported because they failed to meet the benchmark t-stat = 1.96 and *p* < 001.

**FIGURE 2:**
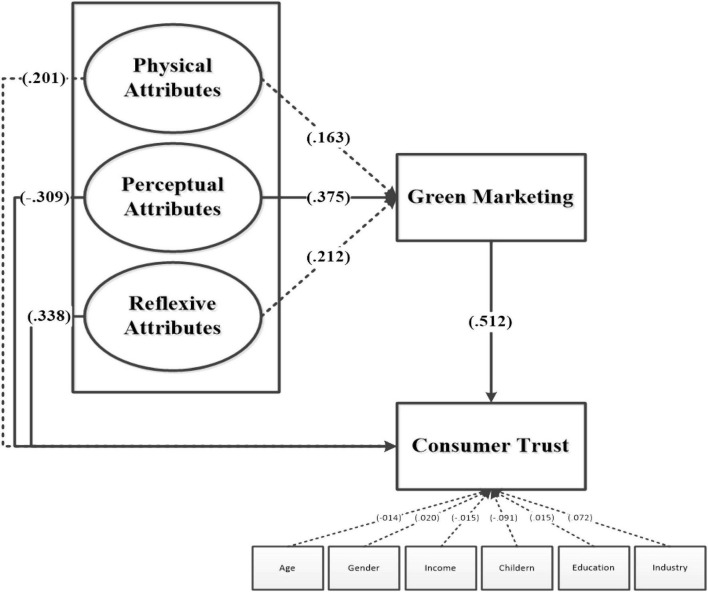
Smart PLS results.

Figure 2 shows the overall results of the proposed model in which we found the negative effect of control variables.

### Indirect Effect

In this study, we checked the indirect effect of product attributes on consumer trust; we followed the recommendation of Zhao et al. (2010) and conducted the Sobel test (Sobel, 1982). The Sobel test results indicate a partial mediating role of green marketing between product attributes and consumer trust (β = 2.584, *p* < 0.001).

### Common Method Bias

In the survey method common method bias (CMB) can be an issue, and to minimize CMB, different organizations including manufacturing, service, and education all over Pakistan were targeted. In Pakistan along with the national language, i.e., Urdu and other regional languages, English is a major language used in daily life. Hence, the questionnaires were prepared in English language and sent to the respondents. We follow Harman (1976) single-factor analysis to calculate CMB. The exploratory factor analysis should be less than 50% to avoid CMB. Results show that the total variance for the single factor was less than 50%, which suggests that CMB was not an issue in the current study (Podsakoff et al., 2012).

## Discussion and Implications

We hypothesized that the physical attributes of a green product have a positive effect on green marketing. Results in Table 5 are insignificant and do not support this hypothesis. Existing research shows that the relationship between physical attributes and green marketing involves a social dilemma in which both the environmental and individual interests are at odds (Gupta and Ogden, 2009).

**TABLE 5:** Structural paths and hypothesis tests.

Hypothesis	Relationship	Standardized regression weights (β)	t-statistics	*P*-value	Result
H1	Physical attributes → Green Marketing	0.163	1.39	0.164	Not supported
H2	Perceptual attributes → Green Marketing	0.375	2.58	0.010*	Supported
H3	Reflexive Attributes → Green Marketing	0.212	1.78	0.075	Supported
H4	Physical Attributes → Consumer trust	0.201	1.421	0.156	Not supported
H5	Perceptual Attributes → Consumer trust	−0.390	2.722	0.007*	Supported
H6	Reflexive Attributes → Consumer Trust	0.338	2.223	0.027*	Supported
H7	Green marketing → Consumer Trust	0.512	4.813	0.000***	Supported

Significant at: *p < 0.05; **p < 0.01; ***p < 0.001 N = 396; T = 1.96 (min) for significance. Resource: Authors.

The results of this study are insignificant because consumers in Pakistan might be involved in a tradeoff situation like purchase decisions regarding physical attributes and green marketing, same as the point of tradeoff that Dawes and Messick (2000) focused on much in their research. The point is that the self-serving motives of individuals influence the purchase decision based on product attributes and green marketing.

The second hypothesis shows a positive influence on the perceptual attributes of green products toward green marketing. Results (Table 5) of this research show that consumers’ perceived attributes of green products are not intended toward green marketing. Gershoff and Frels (2015) in their research concluded that there are varying degrees in which green marketing is affected by perceptual attributes of the product. These three varying degrees are awareness, motivation, and involvement of consumers in making the environment green.

Our research results are consistent with previous studies by Luchs et al. (2010) who in their research conclude that as the perceived performance attributes of a product increases the purchase preferences and intention toward green marketing of consumer decreases. This important contribution of our research can be used as a guide by companies to consider perceptual factors of a green product while devising their marketing strategies.

The findings of this research are also supported by Banerjee and McKeage (1994) results of regular green consumers being motivated by their internal values and assumptions of the currently worsening state of the environment. Such consumers pay more attention to the reflexive attribute of the product to the extent to which they perceived that the consumption of the product would aid in emphasizing their role as a “concerned” consumer. We discussed this relationship, i.e., H3, and it was found to have exhibited a significant relationship between reflexive product characteristics and consumer trust to make a purchase decision about a green product.

However, the findings on this link are still inconclusive in terms of a positive correlation having a role in encouraging purchase behaviors (Laroche et al., 2001). This may have resulted in such marketing objectives being validated by the intrinsic values that one consumer may hold regarding their consumption of such products and convert it into (trust) a motivating purchase behavior (Baldwin, 1993). The unique contribution of this research is the investigation of the largely ignored impact of reflexive green products’ attributes and consumer trust. Chase and Smith (1992) further address this relationship by citing that environmental marketing campaigns have been shaped by research into a consumer’s internal values (both moral and esteem-based), which has shaped the course of 70% of successful green marketing campaigns conducted within the last two decades.

Amyx et al. (1994) studied the construct of green trust among consumers concerning its mediating role in terms of governing their attitudes toward products that have been marketed as being green and found that products that exhibited concern and care for both the consumer and environment in an equal measure were more effective in promoting an eco-friendly outlook among consumers, making them more trustful of the products being marketed as such. Recyclability was also found to have a strong relationship with consumers’ own internal affinity toward green products in terms of recyclable products being considered environmentally responsible on their own merits, and therefore more trustful by potential consumers. This may further be augmented by the perception of their involvement in the ongoing protection of the environment (McCarty and Shrum, 1994).

The last contribution of this research is the significant mediating role of green marketing campaigns between product attributes and consumer trust in influencing sustainable green purchasing behavior. The last hypothesis H8 was validated through the Sobel (1982) test and shows a partial mediating relationship of green marketing practices between green product attributes and consumer trust to influence sustainable green purchasing behavior. Similar to the results of this research, the existing research also exhibits similar results and characteristics. Lyer (1999) posited that the act of eco-labeling and green packaging has enhanced the environmental appeal of products in terms of projecting their purported environmental benefits to the expectations of the consumer. Bleda and Valente (2008) further corroborate these findings by stating that if green marketing initiatives meet consumer expectations of eco-friendliness, it will lead to an enhanced degree of trust on the part of the consumer.

Ginsberg and Bloom (2004) further add the notion of the brand image being augmented by expectations (perceptions) of green performance, which may lead to a higher degree of trust among consumers than if those marketing elements were kept disparate. An element of loyalty may come into action when consumers are deciding among similarly performing products, and eco-trust may prove to be the determinant factor in terms of guiding their purchase decisions (Rahbar and Wahid, 2011).

## Conclusion

This study aimed to investigate the impact of green product attributes on consumer trust i.e., sustainable consumer buying behavior. We also investigated the factors that contribute toward the effective implementation of green marketing campaigns and their intended effect on the development of consumer trust. The quantitative technique was employed to statistically analyze the impact of green marketing between green product attributes and consumer trust, i.e., consumer intentions to purchase green products in Pakistan. Consumer trust is a key prerequisite for establishing a green product market. The results revealed a positive strong correlation between green product attributes, such as perceived and perceptual attributes, and consumer trust to purchase green products. Consumers’ trust is developed by green marketing strategies.

The results of this research will aid in guiding the development of future green marketing campaigns in terms of fine-tuning their focus on the elements that have been noted to have a considerable effect in terms of shaping consumers’ belief (trust) in the merits of such “green” products.

## Data Availability Statement

The raw data supporting the conclusions of this article will be made available by the authors, without undue reservation.

## Author Contributions

KK, FA, and FK wrote the manuscript. MA and SA gathered the data. MA and KK analyzed the data. All authors read and approved the final manuscript.

## Conflict of Interest

The authors declare that the research was conducted in the absence of any commercial or financial relationships that could be construed as a potential conflict of interest.

## Publisher’s Note

All claims expressed in this article are solely those of the authors and do not necessarily represent those of their affiliated organizations, or those of the publisher, the editors and the reviewers. Any product that may be evaluated in this article, or claim that may be made by its manufacturer, is not guaranteed or endorsed by the publisher.
